# Diagnostic accuracy of magnetic resonance imaging for post-radiotherapy recurrent laryngeal cancer: a comparison study with primary laryngeal cancer

**DOI:** 10.1017/S0022215126105052

**Published:** 2026-07

**Authors:** Süleyman Cebeci, Emirhan Akyol, Murat Uçar, Mehmet Arda İnan, M.Mehmet İncesu, Ilayda Kılıç, Mehmet Düzlü, Utku Aydil, Muammer Melih Şahin, Recep Karamert

**Affiliations:** 1 Department of Otorhinolaryngology/ Head and Neck Surgery, Faculty of Medicine, Gazi University, Ankara, Türkiye; 2 Department of Radiology, Faculty of Medicine, Gazi University, Ankara, Türkiye; 3 Department of Medical Pathology, Faculty of Medicine, Gazi University, Ankara, Türkiye

**Keywords:** laryngeal cancer, magnetic resonance imaging, recurrence, radiotherapy

## Abstract

**Objectives:**

Post-radiotherapy tissue changes in laryngeal cancer can hinder accurate imaging assessment of recurrence and tumour extent. We compared pre-operative magnetic resonance imaging staging performance in recurrent laryngeal cancer versus primary laryngeal cancer.

**Methods:**

Retrospective single-centre study of 51 total laryngectomy patients (2012–2021). Magnetic resonance imaging assessments of invasion/extension were compared with histopathology (reference standard) to compute accuracy, sensitivity and specificity.

**Results:**

Magnetic resonance imaging accuracy differed between groups. In the recurrent laryngeal cancer group, accuracy was highest for lymph node metastasis (88.5 per cent) and extra-laryngeal spread (76.9 per cent), whereas it was lowest for cartilage invasion (53.9 per cent) and paraglottic involvement (57.8 per cent). In the primary laryngeal cancer group, magnetic resonance imaging performed best for subglottic and supraglottic extension (76.0 per cent each) and cartilage invasion (72.0 per cent), but showed limited accuracy for extra-laryngeal spread (36.0 per cent) and lymph node metastasis (60.0 per cent).

**Conclusion:**

Magnetic resonance imaging tended to overestimate tumour extent in both groups. It was sensitive overall, but less specific in certain subsites, particularly the paraglottic region. Negative nodal findings may be reassuring, although conclusions regarding nodal status remain limited.

## Introductio

Laryngeal cancer (LC), which is most often squamous cell carcinoma and frequently located in the glottic region, is a common malignancy of the head and neck area.^1^^,^^2^ The recurrence rate among patients with LC is 5–13 per cent for T1 tumours, 25–30 per cent for T2 and 30–50 per cent for T3 and T4.^3^ While recurrence after radiotherapy (RT) is an indication for total laryngectomy, the emergence of more innovative and function-preserving surgical techniques in recent years has improved the quality of life.^4^^,^^5^ Accurate staging with a detailed head and neck examination, including endoscopic and imaging methods, is critical in planning the right treatment.^6^

Pre-treatment staging of head and neck tumours, RT planning and evaluation of response to chemo/RT treatment are often performed with computed tomography (CT) and magnetic resonance imaging (MRI) in combination with positron emission tomography (PET).^7^ Anatomical and tissue changes such as interstitial oedema, thickening of connective tissue, reticulation of subcutaneous fat and deep tissue fat layers, lymphatic tissue atrophy, increased fat density in the pre-epiglottic and paralaryngeal spaces and thickening of laryngeal structures are observed after RT.^8^ This may cause difficulties in detecting local recurrence or tumour involvement and spread by physical examination or imaging techniques.^9^ Therefore, imaging modalities play important roles in post-treatment surveillance with their effectiveness in distinguishing tissue changes after RT and tumour invasion.^10^

Recent studies have aimed to determine the effectiveness of pre-treatment imaging methods in cases of LC.^11^^,^^12^ The majority of these studies focused on the effectiveness of CT, which has consistently demonstrated its importance in staging patients with primary LC (PLC). However, MRI studies have yielded conflicting results, and limited research addresses patients with recurrent LC (RLC).^13^^,^^14^ Therefore, this study investigated the diagnostic accuracy of MRI in the staging of LC patients whose disease relapsed after RT and compared it to the accuracy for patients with PLC.

## Materials and methods

This study was a single-centre retrospective study conducted between June 2012 and June 2021 at the Department of Otorhinolaryngology, Gazi University Faculty of Medicine. The study was performed in accordance with the Declaration of Helsinki and received approval from the Gazi University Faculty of Medicine’s Clinical Research Ethics Committee on 28 June 2021 (Decision No 588) Written informed consent was not obtained due to retrospective design of the study.

### Study population

A total of 156 adult patients (aged >18 years) with pathologically confirmed laryngeal squamous cell carcinoma who underwent total laryngectomy between 2012 and 2021 were retrospectively screened; 36 of these patients experienced recurrent disease following radiotherapy. Inclusion criteria were: (i) pre-operative neck MRI acquired using a standardised protocol (as described in the Radiological Evaluation section), enabling assessment of all predefined anatomic subsites, and (ii) post-operative histopathology specimens adequate for systematic review of the same subsites, sufficiently sampled to evaluate tumour extension in the subglottic, glottic/supraglottic, paraglottic and pre-epiglottic regions, cartilage and extra-laryngeal soft tissues and capable of re-examination. Patients were excluded if MRI quality or protocol was non-standard or incomplete for subsite assessment, if pre-operative MRI was unavailable or if histopathological material was insufficient to evaluate the predefined subsites. Following these criteria, 51 patients were included in the final analysis. Of these, 26 patients who had previously received radiotherapy and underwent salvage total laryngectomy were assigned to the recurrent LC (RLC) group, while 25 patients without prior radiotherapy were included as the PLC group.

Patient data were obtained from the hospital database and pathology-radiology archives. Pre-operative MRI scans and post-operative histopathology were reviewed independently by a head and neck radiologist (15 years’ experience) and a pathologist (10 years’ experience). Both reviewers were blinded to each other’s assessments and to the original reports. Using standardised, predefined anatomical criteria, they evaluated subglottic and supraglottic extension, paraglottic and pre-epiglottic space involvement, thyroid cartilage invasion, extra-laryngeal spread and cervical lymph node metastasis in all included patients.

### Radiological evaluation

All MRI examinations were performed using a 3.0-T system (Siemens MAGNETOM Verio, Erlangen, Germany). T1-weighted images (TR/TE: 360/10 ms), axial T2-weighted images (repetition time/echo time [TR/TE]: 3660/109 ms), fat-suppressed T1-weighted images (TR/TE: 700/10 ms) in axial and sagittal planes, coronal STIR images (TR/TE: 3000/92 ms), diffusion-weighted slices (TR/TE: 8000/60 ms) and fat-suppressed post-contrast T1-weighted images in axial, sagittal and coronal planes (TR/TE: 360/10 ms) were acquired for all patients, alongside apparent diffusion coefficient (ADC) maps.

Soft tissue lesions exhibiting contrast enhancement and diffusion restriction on diffusion-weighted images were considered indicative of involvement. Neoplastic cartilage invasion was defined as a hypoechoic rim with ossification in the outer and inner cortices, accompanied by high signal intensity suggesting the disappearance of intramedullary fat and replacement by lesions with abnormal soft tissue signal intensity. These lesions demonstrated enhanced contrast on T1-weighted post-contrast images, high signal on fat-suppressed T2-weighted images and restricted diffusion on diffusion-weighted images. Post-contrast T1- and T2-weighted images were utilised to differentiate peritumoural inflammation from tumour tissue.

Extra-laryngeal spread was assessed as malignant involvement extending beyond the outer cortex into surrounding muscle and fat planes. When paraglottic fat planes were absent, malignancy was classified as paraglottic involvement. Radiological criteria for malignant nodes included a minimum axis diameter greater than 10 mm, a round or spherical shape and the presence of necrotic or indistinct spiculated nodes.

**Figure 1.**
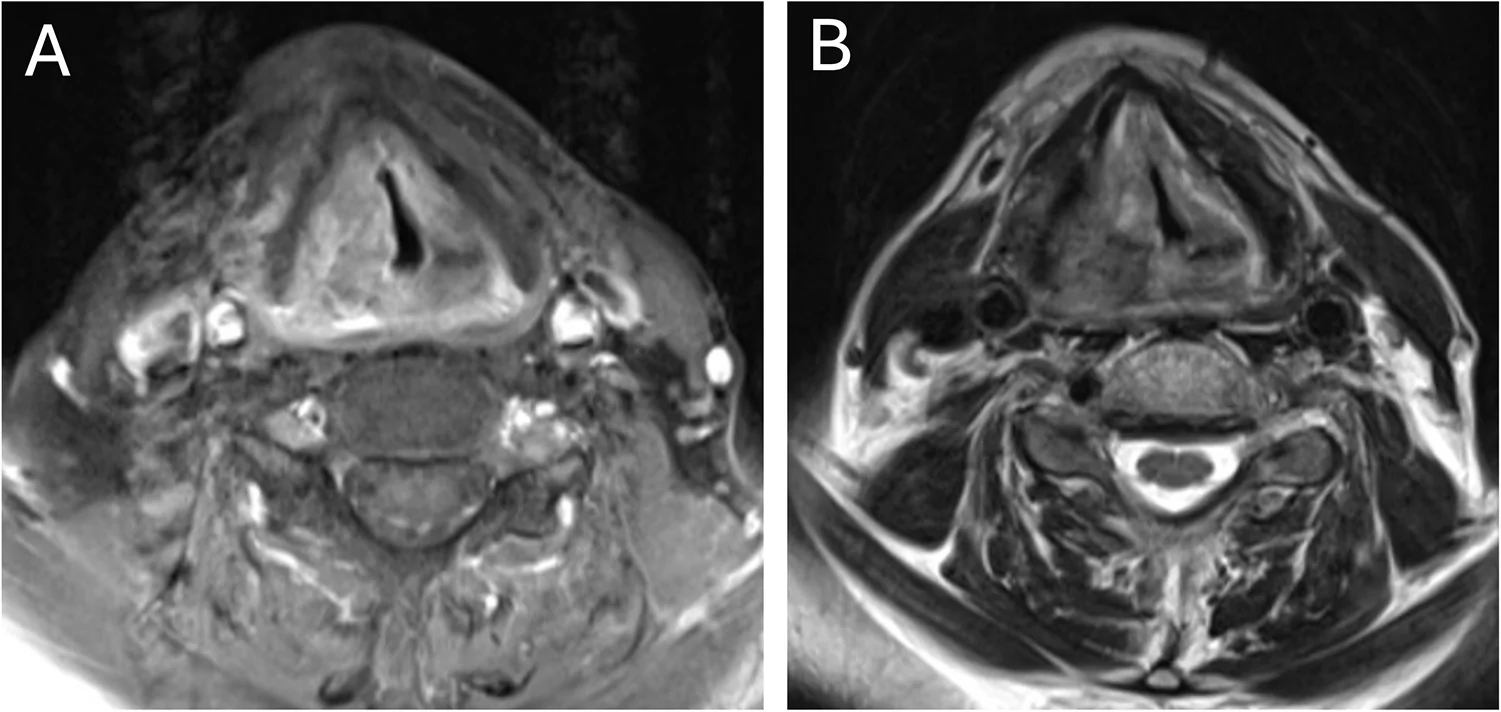
False-positive extra-laryngeal extension on MRI. Extra-laryngeal extension was suspected on MRI, whereas histopathology showed no extra-laryngeal spread. (A) Fat-suppressed post-contrast T1-weighted image. (B) Fat-suppressed T2-weighted image. MRI = magnetic resonance imaging.

**Figure 2.**
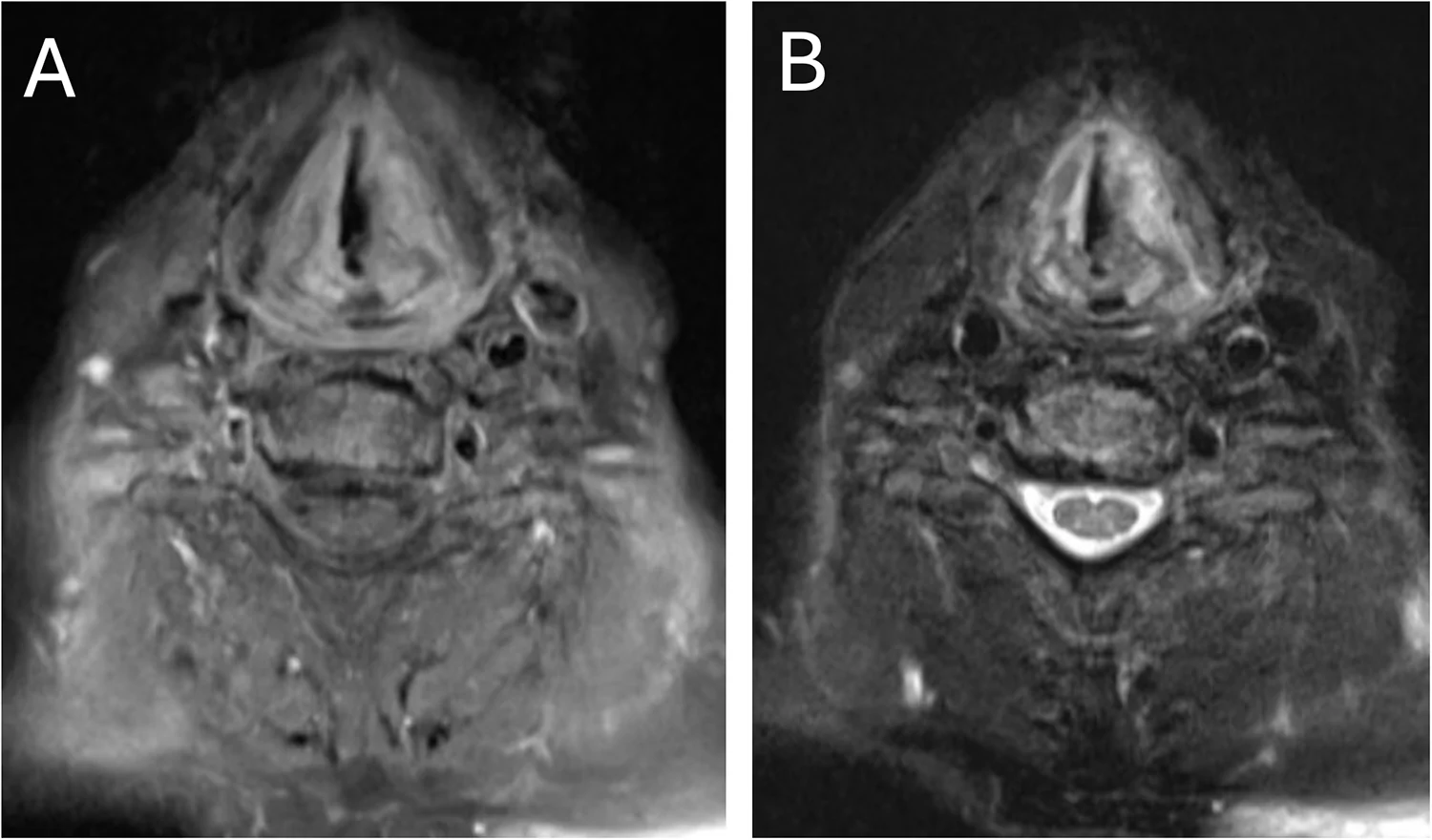
False-positive thyroid cartilage invasion on MRI. Cartilage invasion was suspected on MRI, whereas histopathology showed no thyroid cartilage invasion. (A) Fat-suppressed post-contrast T1-weighted image. (B) Fat-suppressed T2-weighted image. MRI = magnetic resonance imaging.

**Figure 3.**
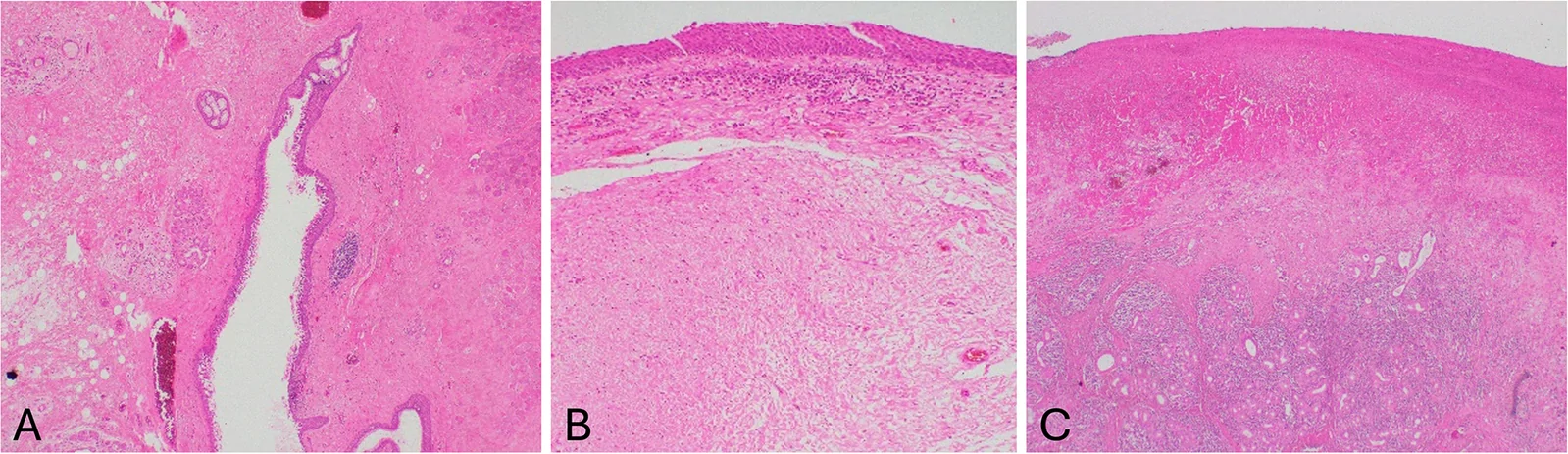
Post-treatment changes mimicking tumour on MRI: histopathological correlation. Hematoxylin and eosin (H&E)–stained sections from regions suspicious for tumour on MRI demonstrate post-treatment changes. (A) Marked fibrotic activity (×40). (B) Minimal subepithelial chronic inflammation with deeper fibrosis (×100). (C) Erosion and necrosis (×40). MRI = magnetic resonance imaging.

MRI images demonstrating extra-laryngeal extension and cartilage invasion are presented in Figures 1 and 2, respectively. Figure 3 illustrates fibrotic activity, chronic inflammation, erosion and necrosis observed on MRI.

### Pathological evaluation

Slides of patient tissue samples stained with hematoxylin and eosin were obtained from the Medical Pathology Department archive, and sections were examined under a light microscope (Olympus BX50) at 12.5×, 40×, 100×, 200× and 400× magnifications (objective lens × 10; ocular lens × 10). Macroscopic findings not obtainable from the sections were documented from the main reports. According to the staging guidelines of the American College of Pathologists and the American Joint Committee on Cancer, updated in 2017 (8^th^ edition), findings from these materials were recorded on Pathological Examination Forms (PEFs). Using the PEFs, findings related to tumour level (subglottic, glottis or supraglottic), cartilage invasion, paraglottic and pre-epiglottic extension, extra-laryngeal extension, differentiation, lymphovascular and perineural invasion status and pTNM staging were re-examined.

### Statistical analysis

Normally distributed data were identified using the Kolmogorov–Smirnov test. Numerical variables were expressed as mean ± standard deviation, while non-normally distributed variables were expressed as median (min–max). Categorical variables were presented as numbers and percentages. Diagnostic performance analysis was performed with receiver operating characteristics (ROC) curves. The diagnostic accuracy of MRI was evaluated based on histopathological results, considered the gold standard for assessing tumour extension. Consequently, the sensitivity, specificity, positive predictive value (PPV), negative predictive value (NPV) and accuracy of the MRI scans were determined in evaluating tumour presence and invasion. A *p* value of less than 0.05 was considered statistically significant. All data were analysed using IBM SPSS Statistics for Windows 20.0 (IBM Corp., Armonk, NY, USA) and MedCalc 11.4.2 (MedCalc Software, Mariakerke, Belgium).

## Results

### Patient characteristics

The study population consisted of 51 patients who had undergone total laryngectomy (mean age: 64.2 ± 9.1 years; range: 43–90 years), including 25 patients with PLC and 26 patients who experienced recurrence following RT and underwent salvage laryngectomy. In the RLC group, the mean time between the last session of RT and relapse detection was 4.0 ± 1.0 months (range: 3–6 months), and the mean time between MRI and surgery was 7.5 ± 1.1 days (range: 5–10 days). The mean age was similar between the PLC and RLC groups (65.3 ± 6.6 years vs. 63.1 ± 11.1 years; *p* = 0.386).

The pathological T stages were predominantly pT3 in both the PLC and RLC groups (64 per cent and 38.5 per cent, respectively), while pathological N stages were primarily classified as N0 (44 per cent and 88.5 per cent, respectively). Eighteen patients in the PLC group had cartilage invasion, 6 had extra-laryngeal spread, 16 had subglottic extension, 18 had supraglottic extension, 17 had paraglottic involvement and 9 had pre-epiglottic involvement. Eleven of the RLC patients had cartilage invasion, 8 had extra-laryngeal spread, 14 had subglottic extension, 14 had supraglottic extension, 15 had paraglottic involvement and 2 had pre-epiglottic involvement. Fourteen patients in the PLC group and three in the RLC group exhibited lymph node metastases. Detailed demographic and clinicopathological findings are presented in Table 1.

**Table 1.** Demographic and clinicopathological findings Table 1 long description.

Variables	All patients *n* = 51	Primary LC *n* = 25	Recurrent LC *n* = 26
Age, years	64.2 ± 9.1	65.3 ± 6.6	63.1 ± 11.1
Gender, n (%)			
Male	50 (98.0)	25 (100.0)	25 (96.2)
Female	1 (2.0)	0	1 (3.8)
pT classification, n (%)			
1	6 (11.8)	2 (8.0)	4 (15.4)
2	10 (19.6)	3 (12.0)	7 (26.9)
3	26 (51.0)	16 (64.0)	10 (38.5)
4	9 (17.6)	4 (16.0)	5 (19.2)
pN classification, n (%)			
0	34 (66.7)	11 (44.0)	23 (88.5)
1	8 (15.7)	6 (24.0)	2 (7.7)
2	5 (9.8)	5 (20.0)	0
3	4 (7.8)	3 (12.0)	1 (3.8)
Cartilage invasion, n (%)	29 (56.9)	18 (72.0)	11 (42.3)
Extra-laryngeal spread, n (%)	14 (27.5)	6 (24.0)	8 (30.8)
Subglottic extension, n (%)	30 (58.8)	16 (64.0)	14 (53.8)
Supraglottic extension, n (%)	32 (62.7)	18 (72.0)	14 (53.8)
Paraglottic involvement, n (%)	32 (62.7)	17 (68.0)	15 (57.7)
Pre-epiglottic involvement, n (%)	11 (21.6)	9 (36.0)	2 (7.7)

*Note:* Data are shown as mean ± standard deviation and number (%).

LC = laryngeal cancer; pT = pathological T classification; pN = pathological N classification.

### Imaging accuracy

True positive and true negative findings from the MRI results, as compared to pathology findings, are presented in Table 2. Among the PLC patients, 16 were classified as false positive for extra-laryngeal invasion, 5 for subglottic invasion, 5 for supraglottic invasion, 8 for paraglottic involvement and 9 for pre-epiglottic involvement. In the RLC patients, the MRI findings overestimated tumour involvement and extension.

**Table 2.** True positive and true negative findings of MRI results according to histopathological results Table 2 long description.

	Primary LC		Recurrent LC	
	Pathological results		Pathological results	
MRI results	Yes	No	Yes	No
Cartilage invasion, n (%)				
Yes	18 (100)	7 (100)	10 (90.9)	11 (73.3)
No	0	0	1 (9.1)	4 (26.7)
Extra-laryngeal spread, n (%)				
Yes	6 (100)	16 (84.2)	7 (87.5)	5 (27.8)
No	0	3 (15.8)	1 (12.5)	13 (72.2)
Subglottic extension, n (%)				
Yes	15 (93.8)	5 (55.6)	10 (71.4)	6 (50.0)
No	1 (6.3)	4 (44.4)	4 (28.6)	6 (50.0)
Supraglottic extension, n (%)				
Yes	17 (94.4)	5 (71.4)	12 (85.7)	8 (66.7)
No	1 (5.6)	2 (28.6)	2 (14.3)	4 (33.3)
Paraglottic involvement, n (%)				
Yes	17 (100)	8 (100)	15 (100)	11 (100)
No	0	0	0	0
Pre-epiglottic involvement, n (%)				
Yes	9 (100)	9 (56.3)	2 (100)	8 (33.3)
No	0	7 (43.8)	0	16 (66.7)
Lymph node metastasis, n (%)				
Yes	12 (85.7)	5 (45.5)	3 (100)	3 (13.0)
No	2 (14.3)	6 (54.5)	0	20 (87.0)

*Note:* Data are shown as number(%).

Abbreviations: LC = laryngeal cancer; MRI = magnetic resonance imaging.

The diagnostic accuracy of MRI in the RLC group was 53.9 per cent for cartilage invasion (sensitivity: 90.9 per cent, 10/11; specificity: 26.7 per cent, 4/15), 76.9 per cent for extra-laryngeal spread (sensitivity: 87.5 per cent, 7/8; specificity: 72.2 per cent, 13/18), 61.5 per cent for subglottic extension (sensitivity: 71.4 per cent, 10/14; specificity: 50.0 per cent, 6/12), 61.5 per cent for supraglottic extension (sensitivity: 71.4 per cent, 10/14; specificity: 50.0 per cent, 6/12), 57.8 per cent for paraglottic involvement (sensitivity: 100 per cent, 15/15; specificity: 0 per cent, 0/11) and 69.2 per cent for pre-epiglottic involvement (sensitivity: 100 per cent, 2/2; specificity: 66.7 per cent, 16/24) (Table 3).

**Table 3.** Accuracy, sensitivity, specificity, PPV and NPV of MRI in primary and recurrent laryngeal cancer groups Table 3 long description.

Parameter	Sensitivity (95% CI)	Specificity (95% CI)	PPV (95% CI)	NPV (95% CI)	ACC (95% CI)
Primary LC					
Cartilage invasion	100 (81.5−100)	0 (0−41.0)	72.0 (72.0−72.0)	-*	72.0 (50.6−87.9)
Extra-laryngeal spread	100 (54.1−100)	15.8 (3.4−39.6)	27.3 (23.6−31.3)	100	36.0 (18.0−57.5)
Subglottic extension	93.8 (69.8−99.8)	44.4 (13.7−78.8)	75.0 (62.3−84.5)	80.0 (34.4−96.8)	76.0 (54.9−90.6)
Supraglottic extension	94.4 (72.7−99.9)	28.6 (3.7−70.9)	77.3 (67.7−84.6)	66.7 (17.6−94.9)	76.0 (54.9−90.6)
Paraglottic involvement	100 (80.5−100)	0 (0−36.9)	68.0 (68.0−68.0)	-*	68.0 (46.5−85.1)
Pre-epiglottic involvement	100 (66.4−100)	43.8 (19.8−70.1)	50.0 (39.4−60.6)	100	64.0 (42.5−82.0)
Lymph node metastasis	85.7 (57.2−98.2)	54.6 (23.4−83.3)	70.6 (54.8−82.6)	75.0 (42.7−92.4)	72.0 (50.6−87.9)
Recurrent LC					
Cartilage invasion	90.9 (58.7−99.8)	26.7 (7.8−55.1)	47.6 (38.9−56.5)	80.0 (34.0−96.9)	53.9 (33.4−73.4)
Extra-laryngeal spread	87.5 (47.4−99.7)	72.2 (46.5−90.3)	58.3 (38.8−75.5)	92.9 (67.0−98.8)	76.9 (56.4−91.0)
Subglottic extension	71.4 (41.9−91.6)	50.0 (21.1−78.9)	62.5 (46.4−76.3)	60.0 (35.5−80.4)	61.5 (40.6−79.8)
Supraglottic extension	85.7 (57.2−98.2)	33.3 (9.9−65.1)	60 (48.8−70.3)%	66.7 (30.6−90.1)	61.5 (40.6−79.8)
Paraglottic involvement	100 (78.2−100)	0 (0−25.5)	57.8 (57.8−57.8)	-*	57.8 (36.9−76.7)
Pre-epiglottic involvement	100 (15.8−100)	66.7 (44.7−84.4)	20.0 (12.4−30.6)	100	69.2 (48.2−85.7)
Lymph node metastasis	100 (29.2−100)	87.0 (66.4−97.2)	50.0 (25.8−74.2)	100	88.5 (69.8−97.6)

* MRI showed paraglottic involvement in all cases thus negative predictive value could not be calculated.

ACC = accuracy; CI = confidence interval; MRI = magnetic resonance imaging; NPV = negative predictive value; PPV = positive predictive value.

The diagnostic accuracy of MRI in the PLC group was 72.0 per cent for cartilage invasion (sensitivity: 100 per cent, 18/18; specificity: 0 per cent, 0/7), 36.0 per cent for extra-laryngeal spread (sensitivity: 100 per cent, 6/6; specificity: 15.8 per cent, 3/19), 76.0 per cent for subglottic extension (sensitivity: 93.8 per cent, 15/16; specificity: 44.4 per cent, 4/9), 76.0 per cent for supraglottic extension (sensitivity: 94.4 per cent, 17/18; specificity: 28.6 per cent, 2/7), 68.0 per cent for paraglottic involvement (sensitivity: 100 per cent, 17/17; specificity: 0 per cent, 0/8) and 64.0 per cent for pre-epiglottic involvement (sensitivity: 100 per cent, 9/9; specificity: 43.8 per cent, 7/16) (Table 3).

MRI detected neck lymph node involvement in 6 RLC patients and 17 PLC patients. The results for 20 RLC patients and 6 PLC patients were true negatives. Pathology revealed neck lymph node involvement in 2 of 6 PLC patients who were initially deemed negative by MRI (Table 2). The diagnostic accuracy, sensitivity and specificity of MRI for neck lymph node involvement were 88.5 per cent (23/26), 100 per cent (3/3) and 87.0 per cent (20/23) in the RLC group and 72.0 per cent (18/25), 85.7 per cent (12/14) and 54.6 per cent (6/11) in the PLC group, respectively (Table 3).

## Discussion

Decisions regarding the treatment and follow-up of LC are typically based on the extent of the tumour and the presence of lymph node involvement in the neck. However, confounding factors such as age and gender play important roles in the course and condition of such diseases.^15^^,^^16^ In this study, MRI overestimated tumour involvement and extension for both PLC and RLC patients with similar age and gender distributions. Although the diagnostic accuracy of MRI for cartilage invasion was higher in the PLC group than in the RLC group, the opposite trend was observed for extra-laryngeal spread. Conversely, MRI demonstrated superior diagnostic accuracy in distinguishing lymph node metastases in the RLC group compared to the PLC group.

A comprehensive clinical, radiological and pathological reassessment is required prior to salvage surgery for patients with recurrent laryngeal carcinoma following RT.^17^ Notably, assessing the extent of tumour spread using imaging modalities is crucial for sound-sparing salvage surgery in cases of RLC. For these patients, accurate assessment of tumour extent can be challenging due to residual inflammatory or functional changes associated with RT. Although necrosis and oedema may be observed histologically during the early period of radionecrosis, the regression of necrosis and tissue healing may enhance the effectiveness of radiology. In areas where invasion is not radiologically evident, tumour infiltration is frequently observed as scattered cell groups without forming significant masses or densities. Within the background of fibrosis resulting from RT, scattered residual tumoural cells may not produce substantial signal changes, thereby complicating the accurate assessment of tumour invasion using imaging modalities for optimal treatment planning.

Previous studies have indicated that CT and MRI exhibit varying degrees of diagnostic accuracy in pre-operative evaluations of LC; however, findings for RLC patients remain limited.^11^^–^^14^ A recent meta-analysis of LC and hypopharyngeal cancers demonstrated that the sensitivity of CT and MRI for primary tumour staging was 66 per cent and 88 per cent, respectively, and the specificity for cartilage invasion was 90 per cent and 81 per cent, respectively.^14^ MRI may yield more false positives for cartilage involvement, potentially leading to overtreatment, while CT may produce fewer true positives, potentially leading to undertreatment.^19^^,^^20^ The present findings are consistent with the results of MRI overestimating tumour involvement and extension.

More false positives were detected in tumour extension measurements, excluding extra-laryngeal spread, in the RLC group compared to the PLC group. It has been suggested that imaging methods yield less accurate results for patients with RLC than PLC.^21^^–^^23^ Previous studies have reported that MRI exhibits high sensitivity (93–96 per cent) and high accuracy (76–84 per cent) in detecting anterior vocal commissure and thyroid cartilage involvement in cases of PLC.^19^^,^^24^ In this study, MRI demonstrated high sensitivity and accuracy for cartilage invasion, subglottic extension and supraglottic extension in PLC patients, while sensitivity and accuracy were lower in RLC patients. This may be related to tissue changes, such as oedema, sclerosis and necrosis, following radiation therapy and tumour invasion.^18^ On MRI, cartilage involvement is observed as a low or medium signal in T1, while it is observed as increased signal intensity in T2 and shows contrast enhancement. These findings demonstrate high sensitivity. Conversely, inflammation and reactive peritumoural changes limit specificity. However, MRI yielded high negative predictive values in excluding cartilage invasion in RLC patients. There are limited reports on the features of tumour invasion into the thyroid cartilage and anterior commissure by imaging method.^25^

For both PLC and RLC patients, MRI showed high sensitivity and NPV in distinguishing extra-laryngeal involvement. However, diagnostic accuracy was greater for RLC patients than for PLC patients. Radiological images revealed a significant increase in tissue oedema secondary to RT. Consequently, evaluating recurrent masses, paraglottic space involvement and cartilage invasion proved challenging (Figures 1 and 2). In RLC patients, a high rate of false positives may be associated with particularly intense fibrosis and increased connective tissue cellularity (Figures 3A,B). Furthermore, ulceration and areas of subepithelial necrosis, often accompanying intense fibrosis, may be misinterpreted as tumours (Figure 3C). These alterations can complicate the differentiation of tumour tissues.^24^ These findings may also contribute to elevated false positives in cases of paraglottic and pre-epiglottic space infiltration, potentially explaining the low specificity.

The infiltration of pre-epiglottic and paraglottic space, independent of cartilage infiltration, elevates the tumour stage to T3. The majority of patients with LC exhibited a histopathological tumour stage of at least T3. Although MRI overestimated pre-epiglottic and paraglottic involvement, no false negatives were detected for any patient. Previous research indicated that the sensitivity of MRI in cases of paraglottic space infiltration ranged from 67 per cent to 95 per cent, while the specificity was low. In cases of pre-epiglottic space infiltration, the sensitivity is 89–100 per cent and the specificity is 80–85 per cent.^26^

Lymph node metastases, which increase the risk of distant metastasis, are important prognostic factors in head and neck cancer.^27^ However, the prognostic role of MRI in lymph node metastasis has yielded inconsistent findings across previous studies, necessitating further investigation. In a study of LC patients, MRI detected 2 of 13 patients with pathological lymph nodes.^24^ Other studies of head and neck squamous cell carcinomas demonstrated high diagnostic accuracy (sensitivity: 72-81 per cent; specificity: 65-87 per cent).^28^^–^^30^ These discrepancies may be related to nodal size, shape, central necrosis and possible extra-capsular infiltration, which are important criteria for detecting nodal metastases by MRI. In addition, MRI may have significant limitations in detecting micrometastases in lymph nodes. Micrometastases can be detected in 25 per cent of cases of clinically N0 neck tumours with diameters most often being about 10 mm.^31^ In our study, while the NPV was 100 per cent for lymph node metastasis among RLC patients, it was 75 per cent for PLC patients. Based on this finding, the absence of lymph node metastasis on MRI may be reassuring in selected cases; however, its implications for elective neck dissection should be interpreted cautiously, particularly in the RLC group, where only three cases were node-positive.

To the best of our knowledge, this study represents the first analysis the accuracy of MRI for each subregion in cases of PLC and RLC. A key advantage was the reevaluation of all MRI and pathology results by a single radiologist and pathologist. The limitations of this retrospective archival study include several factors. First, pathology evaluations were based on re-examination of previously prepared stained slides. Examination of the larynx using whole-organ sections in prospective studies with larger patient cohorts may provide more conclusive results. Furthermore, paraglottic fat planes could not be selected, especially for patients receiving RT, and, in some cases, despite the absence of involvement. For this reason, there might have been an overestimation in our study. Finally, clinical T staging could not be performed due to the ENT clinical protocol and retrospective design. Therefore, the results of endoscopy and surgical specimen histopathology could not be compared.

- **What is already known;**
- Post-radiotherapy changes can mimic tumour on imaging, reducing staging reliability in laryngeal cancer
- MRI is widely used for local staging, but its performance in recurrent disease is less well defined
- **What this paper adds;**
- MRI tends to overestimate tumour extent in both primary and recurrent laryngeal cancer, with limited specificity in selected subsites
- Diagnostic performance differs by setting: cartilage invasion is more accurate in primary disease, whereas extra-laryngeal spread and nodal assessment perform relatively better in recurrent disease
- MRI may support salvage surgery planning (including neck management) when interpreted cautiously alongside clinical and pathological context

## Conclusion

MRI overestimated tumour involvement in both PLC and RLC patients. Although MRI demonstrated high sensitivity across all regions, NPVs varied by location. Nevertheless, MRI generally produced low false-negative rates and reliably excluded invasion beyond the paraglottic region. While MRI-negative nodal findings may be reassuring in selected cases, the limited number of node-positive patients precludes definitive conclusions regarding nodal management, particularly in the RLC group. Consequently, MRI may be useful as part of the overall pre-operative assessment.

## Table 1 Long description

The table summarizes demographic and clinicopathological characteristics for 51 laryngeal cancer patients overall, split into 25 primary and 26 recurrent cases. Average age was in the mid 60s for all groups, and nearly all patients were male (50 of 51). Tumor stage by pathological T category was most often category 3 overall (26 of 51), especially in primary disease (16 of 25), while recurrent cases had fewer category 3 tumors (10 of 26) and relatively more category 2 tumors (7 of 26). Nodal status differed strongly: recurrent cases were mostly node negative (23 of 26), whereas primary cases were less often node negative (11 of 25) and had more node-positive categories. Cartilage invasion was common overall and higher in primary than recurrent disease (18 of 25 vs 11 of 26). Extra-laryngeal spread occurred in about one quarter to one third of patients, slightly higher in recurrent cases (8 of 26) than primary (6 of 25). Subglottic, supraglottic, and paraglottic involvement were frequent in both groups, generally higher in primary cases, while pre-epiglottic involvement was notably higher in primary than recurrent disease (9 of 25 vs 2 of 26). Values are reported as averages with variability for age and as counts with percentages for categorical variables, so comparisons reflect group distributions rather than individual outcomes.

Navigate back to Table 1.

## Table 2 Long description

The table cross-tabulates MRI results (yes or no) against histopathology (yes or no) for primary and recurrent laryngeal cancer across seven disease features. For primary cancer, cartilage invasion and paraglottic involvement show complete agreement: all pathology-positive cases were MRI-positive and there were no pathology-negative cases. Primary extra-laryngeal spread also aligns strongly, with all pathology-positive cases MRI-positive and most pathology-negative cases MRI-negative. For primary subglottic and supraglottic extension, most pathology-positive cases were MRI-positive, but a notable share of pathology-negative cases were MRI-positive, indicating false positives. In recurrent cancer, cartilage invasion and extra-laryngeal spread include some pathology-positive cases with MRI-negative results, suggesting missed findings, and many pathology-negative cases still had MRI-positive results for extra-laryngeal spread. Recurrent pre-epiglottic involvement shows MRI-positive results even when pathology was negative more often than not. Lymph node metastasis differs by group: recurrent cases show MRI-positive results in all pathology-positive cases but also some MRI positives among pathology-negative cases, while primary cases include both missed and false-positive MRI results. Percentages are within each pathology category and some rows have small counts, so apparent differences may be unstable.

Navigate back to Table 2.

## Table 3 Long description

The table reports MRI diagnostic performance for several laryngeal cancer findings in primary versus recurrent groups, listing sensitivity, specificity, positive predictive value, negative predictive value, and overall accuracy with confidence intervals. In primary disease, sensitivity is near perfect for cartilage invasion, extra-laryngeal spread, paraglottic involvement, and pre-epiglottic involvement, while specificity is very low for cartilage invasion and paraglottic involvement and modest for other sites. Primary group accuracy is highest for subglottic and supraglottic extension, and for cartilage invasion and lymph node metastasis, while extra-laryngeal spread has lower accuracy due to low specificity. In recurrent disease, sensitivity remains high for most findings, including perfect sensitivity for paraglottic involvement, pre-epiglottic involvement, and lymph node metastasis, but specificity varies widely. Recurrent extra-laryngeal spread and lymph node metastasis show comparatively strong specificity and higher accuracy than many other recurrent measures. Positive predictive values are generally higher in the primary group for extension findings, while negative predictive values are often high when reported, indicating MRI is better at ruling out some findings than confirming them when specificity is low. Negative predictive value is not provided for paraglottic involvement because MRI identified that involvement in all cases, limiting interpretation for that metric.

Navigate back to Table 3.
